# Cardiorespiratory Fitness and Risk of Cardiovascular Events and Mortality in Middle Age Patients without Known Cardiovascular Disease

**DOI:** 10.3390/jcm12227011

**Published:** 2023-11-09

**Authors:** Amir Aker, Walid Saliba, Fadel Bahouth, Ibrahim Naoum, Barak Zafrir

**Affiliations:** 1 Department of Cardiology, Lady Davis Carmel Medical Center, Haifa 3436212, Israelavinaoum@gmail.com (I.N.); 2 Department of Community Medicine and Epidemiology, Lady Davis Carmel Medical Center, Haifa 3436212, Israel; 3 Faculty of Medicine, Technion—Israel Institute of Medicine, Haifa 3525433, Israel; 4 Department of Cardiology, EMMS Nazareth Hospital, Nazareth 16100, Israel

**Keywords:** exercise stress testing, fitness, cardiovascular disease, outcome, prognosis

## Abstract

Background: Low cardiorespiratory fitness is an established risk predictor for chronic non-communicable diseases. We aimed to investigate the prognostic significance of fitness level on the risk of major adverse cardiac events (MACE, the composite of myocardial infarction, stroke, or all-cause death), in a contemporary cohort of middle-aged subjects without cardiovascular disease. Methods: Retrospective analysis of patients aged 40–60 years without a history of cardiovascular disease. Degree of fitness was determined according to a graded, maximal treadmill exercise stress testing (EST) time achieved, classified into age- and sex-specific quintiles (Q), and categorized as low (Q1), moderate (Q2–Q4) or high (Q5) fitness groups. A multivariable Cox proportional hazard regression model was used to assess the association of fitness level with the risk of MACE. Results: A total of 6836 patients were included, of which 44.5% were women, and the mean age was 52 years. Overall, 289 MACE events occurred during a median follow-up of 7 years. Level of fitness was inversely associated with the presence of cardiovascular risk factors. The multivariable adjusted hazard ratio (95% confidence interval) for MACE was 1.65 (1.12–2.44) and 2.17 (1.40–3.38) in those at moderate and low fitness levels, compared to the high-fitness group (reference), respectively. For each decrease of one metabolic equivalent (MET) unit achieved at peak exercise, the relative risk for MACE increased by 18%. The association between low fitness and MACE was not modified by other risk factors (P-for-interaction non-significant). Conclusions: Low fitness level, as captured by a maximal treadmill EST, is an independent risk predictor for MACE among middle-age individuals without known cardiovascular disease. The association of low fitness with high burden of cardiometabolic risk factors highlight the importance of lifestyle intervention in this patient population.

## 1. Introduction

Regular physical activity is associated with higher levels of cardiorespiratory fitness and a lower risk for developing chronic medical illnesses, including cardiovascular disease and mortality [1]. These health benefits were observed globally in both low- and high-income countries and in those with higher recreational and non-recreational physical activity [2]. Self-reported and measured physical fitness is a strong predictor of health status. Particularly, cardiorespiratory fitness estimated by exercise tests has been consistently shown to be a strong prognostic measure for chronic disease outcomes and mortality [3]. Graded exercise stress testing (EST) is a commonly available, non-invasive tool often used as a diagnostic test for the assessment of myocardial ischemia and the risk for coronary artery disease. It provides data on vital signs and electrocardiographic responses to exercise and recovery. However, it also evaluates exercise capacity, which by itself comprises important prognostic information [3,4]. The degree of cardiorespiratory fitness captured by EST was repeatedly shown to independently predict adverse outcomes, especially mortality, beyond electrocardiographic changes, and across different populations [5]. In two recent updated meta-analyses including more than 30 observational studies, a high level of cardiorespiratory fitness, objectively assessed by EST, was strongly and independently associated with reduced risk of mortality [6,7]. Of note, most of the study cohorts in these meta-analyses included subjects performing baseline EST before the year 2000. The aim of the present study was to investigate the role of cardiorespiratory fitness, as measured according to the exercise capacity achieved at a graded maximal treadmill EST, in predicting a composite outcome event of myocardial infarction, stroke or all-cause death. We analyzed a contemporary cohort of middle-aged individuals without a history of cardiovascular disease, a population in which fitness level may provide important public health implications [1,8].

## 2. Materials and Methods

### 2.1. Study Population

**Figure 1:**
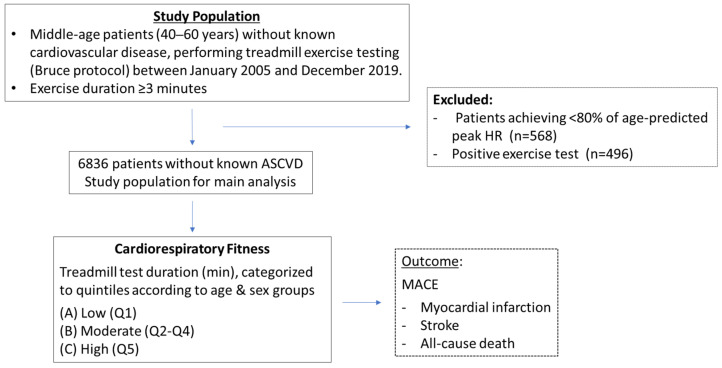
Study population outline.

This study is a retrospective analysis of treadmill EST performed at the Cardiology department in Carmel Medical Center, Haifa, Israel, between the period of January 2005 and December 2019. Only patients between the ages of 40 and 60 years without a history of cardiovascular disease, performing EST by the Bruce protocol, were included in the study. Patients in whom exercise duration was less than 3 min as well as those achieving under 80% of age-predicted heart rate at peak exercise, were excluded from the study population. In addition, patients in whom EST was defined as “positive” due to symptoms or electrocardiographic changes suggestive of ischemia were excluded. The study population outline is presented in Figure 1. 

The primary study endpoint was major adverse cardiovascular events (MACE), defined as a composite of myocardial infarction, stroke (ischemic and hemorrhagic) or all-cause death during long-term follow-up. Data on myocardial infarction and stroke were retrieved from the Clalit Health Services Health Maintenance Organization (HMO) hospitalization database and were defined as primary discharge with ICD-9 code (410.xx) for myocardial infarction and ICD-9 codes (431, 433.x1, 434.x1, 436) for stroke. Data on vital status were retrieved from the Ministry of the Interior. Cohort participants were followed up on until the first occurrence of the study outcome (MACE) was reached, or at the end of the follow-up in February 2021, whichever came first. Demographic data, risk factors, comorbidities, and drug treatment at the timepoint of exercise testing were retrieved from a computerized database of the HMO. The study was approved by the Carmel Medical Center Ethics Committee with waiving of the need for individual patient consent due to the retrospective nature of the study.

### 2.2. Exercise Stress Testing and Fitness Level

Maximal graded treadmill EST was accomplished using a Bruce protocol in all patients [3]. EST was performed mainly as a diagnostic test, both as a screening tool as part of a periodic health examination, or as part of the evaluation of ischemia. Participants were instructed not to take beta-blockers in the morning of the test and to make a maximal effort during a graded incremental exercise. The software program of the treadmill (General Electric, Marquette 2000, Boston, MA, USA) calculated metabolic equivalent (MET) units based on a continuous calculation which was updated every few seconds during the first 2 min of each 3 min stage of the Bruce protocol. During the third minute, the value remained unchanged. We used the maximal value provided by the system software at peak exercise for the METs achieved. Heart rate and blood pressure were measured at rest and at the second minute of each Bruce stage during exercise and recovery. 

**Figure 2:**
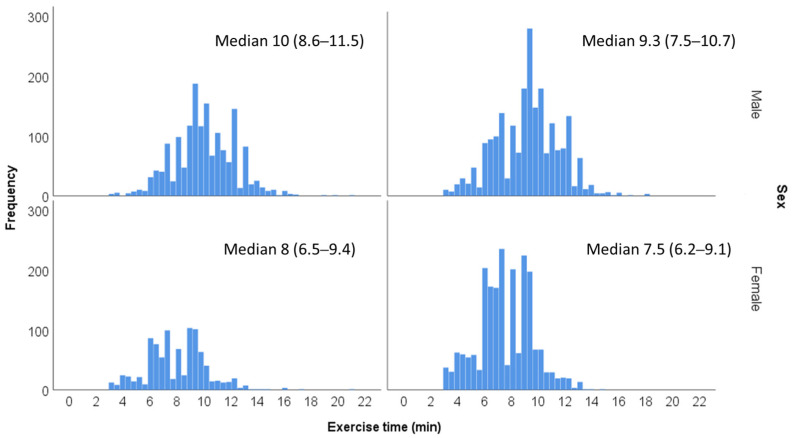
Distribution of exercise time duration according to age and sex groups.

Fitness level was determined according to the overall time of exercise achieved by each patient, performed according to the Bruce protocol. Treadmill exercise time during EST was shown to be in good correlation with maximal oxygen uptake in previous studies [9]. Exercise time duration was categorized according to sex and age groups (40 to 49 and 50 to 59 years) (Figure 2). Each of the 4 age/sex groups were divided into quintiles (Q1–Q5) that were summed and combined into 3 fitness groups: low fitness (Q1, *n* = 1372), moderate fitness (Q2–Q4, *n* = 4127) or high fitness (Q5, *n* = 1337). Exercise capacity was additionally assessed as a quantitative measure according to the METs achieved at peak exercise.

### 2.3. Data Analysis

Continuous data are reported as means and standard deviation or median and interquartile range (IQR), and categorical variables are presented as numbers and percentages. Demographics, comorbidities, medications, and exercise testing parameters are presented according to the 3 fitness subgroups. The one-way analysis of variance (ANOVA) test was used to compare continuous variables and Pearson’s Chi-square test to compare categorical variables. Histograms were used to visually display the distribution of exercise duration in the study population according to age and sex groups. The number of events during the follow-up period and incidence rates per 1000 person-years were calculated according to the fitness categories. Cox proportional hazard regression models were used to assess the association between fitness groups and time to MACE endpoint and its components, estimating adjusted hazard ratios (HRs) and 95% confidence intervals (CI), with the group of patients of high fitness serving as the reference category. Adjustment was made for age and sex, as well as to socioeconomic status, hyperlipidemia, hypertension, smoking, obesity, diabetes mellitus, chronic kidney disease, blood-pressure-lowering drugs, beta blockers, and statins. Exercise capacity was also analyzed as a continuous variable, according to peak METs achieved during exercise, with similar adjustments. Kaplan–Meier plots were used to estimate the cumulative incidence of MACE over time according to fitness level categories, with comparison between curves performed using the log-rank test. Subgroup analysis was performed to evaluate the interaction between individual risk factors and fitness level, regarding the risk for MACE, with calculation of P-for-interaction and graphical presentation by Forest plots. 

The results were considered statistically significant when the 2-sided *p*-value was < 0.05. SPSS statistical software version 25.0 (SPSS, Chicago, IL, USA) and MedCalc^®^ Statistical Software version 19.7.2 (MedCalc Software Ltd., Ostend, Belgium) were used to perform all statistical analyses.

## 3. Results

### 3.1. Patients Characteristics

Included in the analysis were 6836 patients aged 40–60 years, without known cardiovascular disease, undergoing maximal treadmill EST by the Bruce protocol. Mean age was 52 ± 6 years, and 44.5% were women. Baseline clinical characteristics and EST parameters according to fitness categories are presented in Table 1. METs achieved at peak exercise increased across fitness groups, from 7.7 to 10.7 and 13.7 units in low-, moderate-, and high-fitness patients, respectively (*p* < 0.001). Resting heart rate and systolic blood pressure (at rest and peak exercise) were lower in those at the higher level of fitness. A graded inverse association was observed between patient fitness level and the prevalence of cardiovascular risk factors and comorbidities. Compared to the moderate- and high-fitness groups, those at the low fitness level had significantly higher rates of obesity, hypertension, and diabetes. They were also more commonly classified as low socioeconomic status, with higher rates of smoking and chronic obstructive pulmonary disease (Table 1).

### 3.2. Fitness Level and Adverse Outcomes

**Figure 3:**
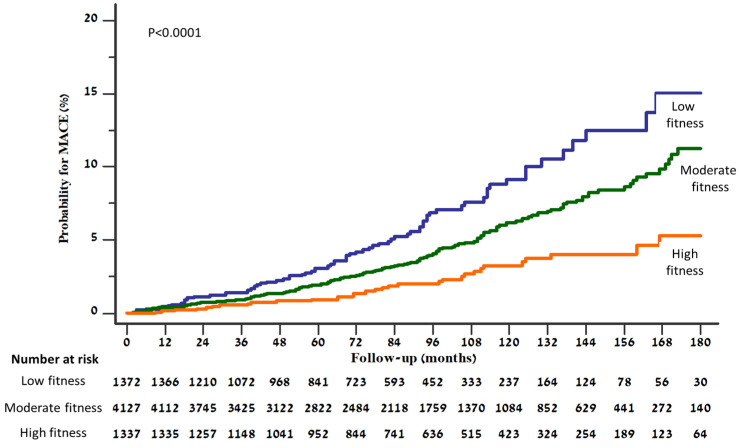
Kaplan–Meier curves presenting cumulative risk for developing MACE, according to fitness level.

During a median follow-up period of 85 months (IQR 49–121 months), 130 (1.9%) patients experienced myocardial infarction, 53 (0.8%) stroke, and 118 (1.7%) died. Overall, the first event of MACE occurred in 289 (4.2%) patients during follow-up. A Kaplan–Meier plot displaying the distribution of time to MACE stratified by categories of fitness levels is presented in Figure 3, showing a graded increment in the risk for MACE with the decrease in fitness level (log rank *p* < 0.0001). 

The incidence rate of MACE and its components per 1000 person-years increased with the decrease in fitness level (Table 2). Compared to patients of high fitness (reference group), the HR for MACE was 1.65 (1.12–2.44) and 2.17 (1.40–3.38) in those of moderate and low fitness, respectively, after multivariable adjustment that included major risk factors, comorbidities, and drug therapy (Table 2). For each decrease of 1-MET achieved at peak exercise, the relative increase in the adjusted HR for MACE was 18%; the relative increase in the risk for all-cause death was 29%, and for myocardial infarction or stroke 13%. 

**Figure 4:**
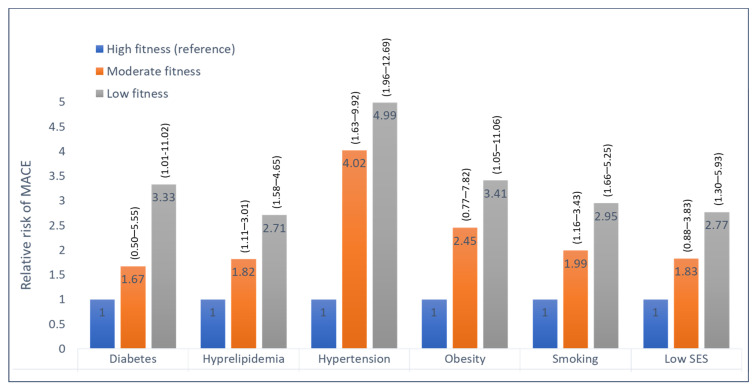
Hazard ratios for MACE among subjects with various risk factors, according to fitness level. High fitness is the reference group (hazard ratio 1); numbers in parentheses are 95 percent confidence intervals for the relative risks; SES, socioeconomic status.

**Figure 5:**
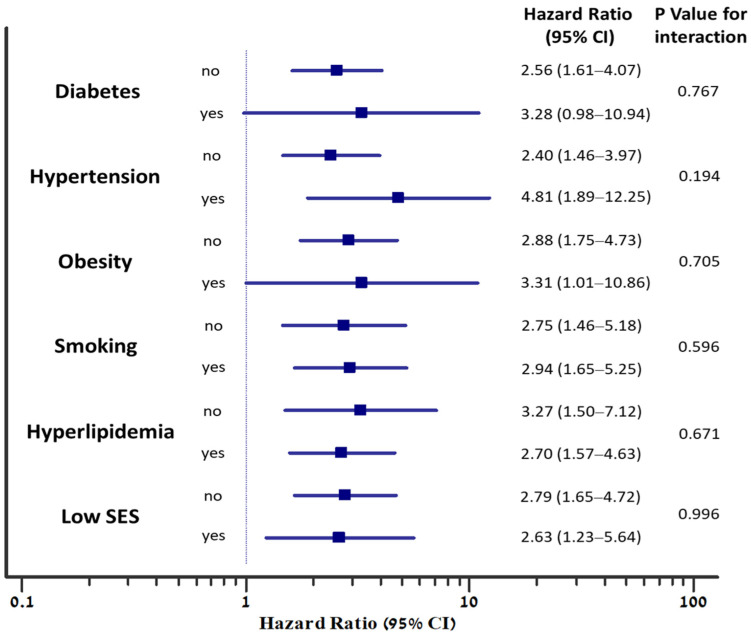
The association between low fitness and the risk for MACE, according to the presence of individual risk factors. Hazard ratios for MACE are adjusted to age and sex. P-for-interaction represents the interaction between the risk factor and fitness level (low compared to high), regarding the risk for MACE; SES, socioeconomic status.

The graded inverse association between fitness level and MACE was observed individually among subjects with each of the traditional cardiovascular risk factors (Figure 4). In addition, no significant interaction was noted between the presence of each of the risk factors (diabetes, hypertension, obesity, smoking, hyperlipidemia, or low socioeconomic status) and low fitness, regarding the risk for MACE (P-for-interaction non-significant), (Figure 5). 

## 4. Discussion

The present study provides further evidence regarding the strong inverse and graded association between cardiorespiratory fitness and future risk for adverse cardiovascular events, in middle-age adults without a history of cardiovascular disease. Fitness was objectively quantified according to the overall time of exercise performed and peak METs achieved, during a maximal treadmill EST using a Bruce protocol. We have shown that low fitness is associated not only with mortality, but also with increased risk for myocardial infarction and stroke, with more than twice the adjusted risk for MACE compared to high-fitness patients and 18% relative increase in risk per 1-MET decrease in exercise capacity. Although our cohort was composed of middle-age adults without known cardiovascular disease, a relatively high burden of cardiovascular risk factors was observed. Low fitness was particularly related to cardiometabolic features, including obesity and diabetes. Nevertheless, the excess risk for cardiovascular events and mortality associated with low fitness was independent of traditional risk factors.

EST is widely available and has been typically used for the evaluation of exercise-related symptoms and assessment of myocardial ischemia [3]. However, apart from the diagnostic value of EST, aerobic exercise capacity measures have significant prognostic importance [4]. In an older meta-analysis of 33 eligible studies of healthy populations, a higher level of maximal aerobic capacity amounting to a 1-MET increase was associated with a 13–15% risk reduction in all-cause mortality or cardiovascular disease [5]. The current study results are consistent with this observation, displaying an 18% relative increase in MACE per 1-MET decrease in exercise capacity. More recently, two large meta-analyses have reported a pooled relative risk for all-cause mortality of 0.88 and 0.89, respectively, per 1-MET increase in cardiorespiratory fitness [6,7]. Regarding cardiovascular disease prevention, the protective effects of physical fitness have been demonstrated particularly by self-reported physical activity questionnaires and more objectively by studies analyzing exercise capacity measures obtained from EST [1] and maximal oxygen uptake evaluated by cardiopulmonary exercise testing (CPET) [10]. Our findings extend the available literature on the independent predictive value of exercise capacity on both cardiovascular disease, mortality, and their composite endpoint, in a contemporary primary prevention cohort of people of middle age, a population in which fitness may have highly important public health implications.

The effect of low cardiorespiratory fitness on the risk for subsequent cardiovascular events was previously suggested to be indirect, moderated through higher metabolic risk [11]. Although we have observed a higher cardiometabolic risk profile in low-fitness patients, the presence of risk factors and comorbidities did not significantly modify the prognostic effect of low fitness on MACE. Previous reports strengthen this observation. In a long-term follow-up population-based study, low aerobic capacity was associated with increased mortality rates, independent of traditional risk factors, during more than 40 years of follow-up [12]. In subjects without prior cardiovascular disease enrolled in the Cooper Center Longitudinal Study, the addition of fitness to a traditional risk factor model resulted in significant net reclassification improvement in risk estimates for cardiovascular mortality [13]. Similarly, in participants of the prospective UK Biobank project, cardiorespiratory fitness, as assessed by submaximal EST, was shown to improve mortality risk prediction beyond conventional risk factors [14]. Past studies have also demonstrated that cardiorespiratory fitness provides additive prognostic information beyond that obtained from standard risk-score calculators and computerized tomography (CT) coronary artery calcium scoring, increasing the accuracy of prediction of cardiovascular disease and mortality [15,16]. Although reduced physical fitness is evidently associated with cardiometabolic risk factors, the interplay between alteration in metabolic profile, cardiorespiratory fitness and future adverse clinical outcomes is not entirely clear and is a subject for further research. Nevertheless, the protective effects of exercise on cardiovascular health are apparently beyond the modification of traditional risk factors. In response to routine exercise, cardiac remodeling with cellular and molecular adaptations occurs, influencing the structure, function, and electrical stability of the heart [17]. In addition, vascular adaptations develop with improved endothelial function and increase in compliance of conductive vessels. Exercise also enhances parasympathetic tone, augments heart-rate variability, and improves baroreflex and autonomic functions [18]. These pathophysiological mechanisms may contribute to the inverse association between fitness and cardiovascular disease. 

Our results, displaying an independent, graded, inverse association between level of fitness and future development of MACE, have important implications. In the current era, in which technology advances are changing the way people live and work, both occupation-related and leisure-time physical activity rates are consistently reduced, and most adults do not meet the aerobic component of physical activity guidelines [19]. Therefore, promoting the benefits of exercise and high cardiorespiratory fitness is particularly important in the primary prevention context of middle-aged adults who are yet to have evidence of cardiovascular disease. In addition, despite its well-established prognostic value, cardiorespiratory fitness is not incorporated in risk assessment tools and prediction models and is not routinely addressed in the healthcare clinical setting [20]. As the cumulative evidence on the health benefits of physical activity and exercise is overwhelming, measures of fitness should be considered as “vital signs”, and cardiorespiratory fitness assessment and improvement plans should be promoted and integrated into routine clinical practice of all health professionals [1].

Several potential limitations should be considered when interpreting this study. First, although the study population was composed of middle-aged adults without a history of cardiovascular disease, the prevalence of traditional cardiovascular risk factors was relatively high. Therefore, the study results may not represent a healthy population, and the generalizability of the findings to other populations may be limited. Second, almost half of the study population were women. Although we have taken into consideration sex differences in both the study design and analysis, it is important to note that physical and physiological differences exist between women and men in terms of exercise performance, response, and adaptations to physical activity [21]. Third, some lifestyle habits such as dietary patterns and alcohol consumption were not evaluated in the current study and may be associated with adverse outcomes [22]. Fourth, additional variables that may have an influence on exercise capacity, such as musculoskeletal or neurological disorders, were not assessed and may have possible effects on residual confounding. However, we did exclude individuals who did not perform significant effort (less than 3 min of exercise and/or under 80% of age-predicted peak heart rate), and those whose EST was suggestive of ischemia. Fifth, exercise-capacity data were estimated based on the time of exercise performed and peak METs achieved during a graded maximal EST. Although direct measurement of peak oxygen uptake by CPET may be a more accurate and reproducible method for assessing cardiorespiratory fitness [10], it is not widely available in the setting of routine clinical medicine. Finally, we did not have information on causes of death, and therefore all-cause and non-cardiovascular mortality were analyzed. Moreover, due to the observational cohort design, causality between cardiorespiratory fitness and adverse outcomes cannot be determined.

## 5. Conclusions

The level of cardiorespiratory fitness, as estimated according to exercise capacity achieved during a graded maximal EST, provides significant prognostic value in middle-aged individuals without a history of cardiovascular disease. Despite its association with comorbidity and the presence of cardiometabolic risk factors, low fitness is an independent predictor for future cardiovascular disease and mortality during a median follow-up of 7 years. These findings suggest that low fitness can be used as a measure to identify high-risk middle-aged subjects without a history of cardiovascular disease in need of intensive primary prevention strategies, focusing on both increased physical activity and cardiorespiratory fitness as well as improved cardiometabolic risk profile, aiming to prevent the morbidity and mortality associated with cardiovascular disease.

## Figures and Tables

**Table 1:** Baseline clinical characteristics.

Variable	Overall (*n* = 6836)	Low Fitness (*n* = 1372)	Moderate Fitness (*n* = 4127)	High Fitness (*n* = 1337)	*p* Value
Age (years)	51.9 ± 5.9	52.3 ± 5.9	51.9 ± 5.9	51.4 ± 5.9	0.001
Sex (Female)	3040 (44.5%)	610 (44.5%)	1828 (44.3%)	602 (45%)	0.896
Socioeconomic status (Low)	2372 (34.7%)	655 (47.7%)	1429 (34. 6%)	288 (21.5%)	<0.001
Body Mass Index (BMI)	27.9 ± 4.9	30.4 ± 5.6	27.9 ± 4.6	25.2 ± 3.4	<0.001
Obesity	1905 (27.9%)	661 (48.2%)	1147 (27.8%)	97 (7.3%)	<0.001
Current smoking	2973 (43.5%)	688 (50.1%)	1787 (43.3%)	498 (37.2%)	<0.001
Hyperlipidemia	3506 (51.3%)	805 (58.7%)	2104 (51%)	597 (44.7%)	<0.001
Hypertension	1947 (28.5%)	543 (39.6%)	1177 (28.5%)	227 (17%)	<0.001
Diabetes mellitus	860 (12.6%)	311 (22.7%)	482 (11.7%)	67 (5%)	<0.001
Chronic Kidney Disease	79 (1.2%)	23 (1.7%)	44 (1.1%)	12 (0.9%)	0.115
COPD	100 (1.5%)	37 (2.7%)	53 (1.3%)	10 (0.7%)	<0.001
Beta blockers	915 (13.4%)	267 (19.5%)	535 (13%)	113 (8.5%)	<0.001
Anti-hypertensive drugs	2086 (30.5%)	584 (42.6%)	1245 (30.2%)	257 (19.2%)	<0.001
Statins	2176 (31.8%)	516 (37.6%)	1314 (31.8%)	346 (25.9%)	<0.001
Exercise testing parameters					
Exercise duration (min)	8.8 ± 2.4	5.7 ± 1.3	8.8 ± 1.4	11.8 ± 1.7	<0.001
Resting heart rate (BPM)	81 ± 13	87 ± 14	81 ± 13	75 ± 12	<0.001
Peak heart rate (bpm)	159 ± 13	154 ± 13	158 ± 13	163 ± 12	<0.001
Peak METs	10.7 ± 2.4	7.7 ± 1.3	10.7 ± 1.4	13.7 ± 1.7	<0.001
Systolic BP—rest	118 ± 15	125 ± 15	118 ± 15	112 ± 14	<0.001
Systolic BP—peak	162 ± 22	167 ± 24	162 ± 21	156 ± 21	<0.001

BPM, beats per minute; COPD, chronic obstructive pulmonary disease; METs, metabolic equivalents.

**Table 2:** Occurrence of cardiovascular events and/or death during follow-up, according to fitness level.

Fitness Level	Proportion with Event	Rate Per 1000 Person-Years	Age- and Gender- Adjusted HR	Multivariable * Adjusted HR
MACE				
High Fitness	32/1337 (2.4%)	3.02	Ref.	Ref.
Moderate Fitness	181/4127 (4.4%)	5.91	1.98 (1.36–2.88) *p* < 0.001	1.65 (1.12–2.44) *p* = 0.011
Low Fitness	76/1372 (5.5%)	8.45	3.04 (2.01–4.61) *p* < 0.001	2.17 (1.40–3.38) *p* < 0.001
For each decrease in 1-MET			1.21 (1.15–1.28) *p* < 0.001	1.18 (1.12–1.25) *p* < 0.001
All-cause Death				
High Fitness	10/1337 (0.7%)	0.94	Ref.	Ref.
Moderate Fitness	71/4127 (1.7%)	2.28	2.47 (1.27–4.79) *p* = 0.007	2.32 (1.15–4.67) *p* = 0.019
Low Fitness	37/1372 (2.7%)	4.06	4.75 (2.36–9.57) *p* < 0.001	4.45 (2.13–9.33) *p* < 0.001
For each decrease in 1-MET			1.30 (1.19–1.42) *p* < 0.001	1.29 (1.18–1.41) *p* < 0.001
Myocardial Infarction or Stroke				
High Fitness	23/1337 (1.7%)	2.17	Ref.	Ref.
Moderate Fitness	113/4127 (2.7%)	3.69	1.72 (1.10–2.69) *p* = 0.018	1.63 (1.03–2.57) *p* = 0.037
Low Fitness	43/1372 (3.1%)	4.78	2.38 (1.43–3.96) *p* < 0.001	1.96 (1.16–3.31) *p* = 0.012
For each decrease in 1 MET			1.16 (1.09–1.24) *p* < 0.001	1.13 (1.06–1.21) *p* = 0.001

* Adjusted for age, sex, socioeconomic status, hyperlipidemia, hypertension, smoking, obesity, diabetes mellitus, chronic kidney disease, blood-pressure-lowering drugs, beta blockers, statins. HR, hazard ratio; MACE, major adverse cardiovascular events (myocardial infarction, stroke or all-cause death); MET, metabolic equivalent.

## Data Availability

The data of this study are available from Clalit Health Services but restrictions apply to the availability of these data, which were used under the license for the current study and are not publicly available.
